# 502 Demographic Variability and Low Utilization of Burn Peer Support: An Analysis of the Current Landscape

**DOI:** 10.1093/jbcr/iraf019.131

**Published:** 2025-04-01

**Authors:** Dania Johnson, Kara McMullen, Elizabeth Flores, Caitlin Orton, Jennifer Bell-De Paz, Jill Sproul, Cindy Rutter, Haig Yenikomshian

**Affiliations:** University of California Keck School of Medicine; University of Washington; University of Southern California / LA General Burn Unit; University of Washington; University of Texas Southwestern; Phoenix Society for Burn Survivors; Southern California Burn Model System; University of California Keck School of Medicine

## Abstract

**Introduction:**

Social support is crucial for burn survivors in managing the challenges of significant injuries. Peer support, where individuals share experiences related to health or life circumstances, is increasingly popular. It is associated with better coping and enhanced social connectedness, making it a valuable part of rehabilitation. Identifying patterns and gaps in care is paramount for a cohesive rehabilitation process. This study analyzes trends in peer support participation and demographic characteristics of those engaging in peer support during their recovery.

**Methods:**

This is a retrospective analysis of adult burn survivors over the age of 18 from 2014-2024 participating in a multicenter longitudinal patient reported outcome database. Participants were queried at 6 and 12 months post-injury about whether they had spoken with other burn survivors for support regarding their burn injuries since the last questionnaire. Participants were divided into two groups at 12 months (peer support/no peer support), and their demographics and injury characteristics were compared. Wilcoxon-Mann-Whitney tests were used for continuous variables due to non-parametric nature of the data, while Chi-square tests were used to test for differences between the populations. To adjust for multiple comparisons, significance level was corrected to 0.0025 using Bonferroni’s method.

**Results:**

Data from 1,123 participants met inclusion criteria for this study. Participation rates of peer support at 6, 12, and 24-months post-injury were 17% (n=843), 15% (n=700), and 15% (n=609) respectively. At 12 months, participants receiving peer support had significantly larger TBSA burned (27+/- 23.3% vs. 16+/- 16.4%, p < 0.001), longer hospital stays (41+/- 41.6 days vs. 25+/- 31.5 days, p < 0.001), and higher education levels (68% > than a high school education vs. 50%, p= 0.001) than those not receiving support. They were more likely to have head/face/neck burns (65% vs. 48%, p= 0.002) and fire/flame etiologies (76% vs. 55%, p< 0.001). Both groups were similar in age (46+/-16.5 years vs. 48+/-16.6 years, p=0.372) with a non-Hispanic/Latino predominance (17% Hispanic/Latino, 83% non-Hispanic/Latino vs. 22% Hispanic/Latino, 78% non-Hispanic-Latino, p=0.214).

**Conclusions:**

Only a small percentage of burn survivors received peer support, with similar rates at 6, 12, and 24 months post-injury. Those engaging in peer support had larger burns, longer hospital stays, and higher education levels, while non-engagers had smaller burns and lower education levels. Efforts should focus on enhancing peer support engagement by addressing barriers like readiness, comfort, and education. Exploring alternative strategies for delivering peer support is essential to improve this vital resource.

**Applicability of Research to Practice:**

This study aims to identify patients underutilizing peer support, highlighting an opportunity to evaluate how effectively resources are meeting their needs.

**Funding for the Study:**

The contents of this abstract were developed under a grant from the National Institute on Disability, Independent Living, and Rehabilitation Research (NIDILRR grant number 90DPBU0007). NIDILRR is a Center within the Administration for Community Living (ACL), Department of health and Human Services (HHS). The contents of this abstract do not necessarily represent the policy of NIDILRR, ACL, or HHS, and you should not assume endorsement by the Federal Government.

